# SARS-CoV-2 Infection: Modulator of Pulmonary Embolism Paradigm

**DOI:** 10.3390/jcm10051064

**Published:** 2021-03-04

**Authors:** Mohammad Suhail Akhter, Hassan A. Hamali, Abdullah A. Mobarki, Hina Rashid, Johannes Oldenburg, Arijit Biswas

**Affiliations:** 1 Department of Medical Laboratory Technology, Faculty of Applied Medical Sciences, Jazan University, Jazan 45142, Saudi Arabia; makhter@jazanu.edu.sa (M.S.A.); hhamali@jazanu.edu.sa (H.A.H.); abMobarki@jazanu.edu.sa (A.A.M.); 2 Department of Pharmacology and Toxicology, College of Pharmacy, Jazan University, Jazan 45142, Saudi Arabia; hzehqeer@jazanu.edu.sa; 3 Institute of Experimental Haematology and Transfusion Medicine, University Clinic Bonn, 53127 Bonn, Germany; Johannes.Oldenburg@ukbonn.de

**Keywords:** COVID-19, pulmonary embolism, inflammation, thrombosis

## Abstract

Pulmonary embolism (PE) is a life-threatening complication arising from venous thromboembolism with a difficult diagnosis and treatment and is often associated with increased mortality and morbidity. PE had a significantly low incidence prior to the COVID-19 epidemic. This condition saw a sharp surge during the COVID-19 pandemic, indicating an evident viral influence on PE’s pathophysiology in COVID-19 patients. The hypercoagulable state induced by the viral load seems to be the major contributor, and the classical causative factors seem to play a lesser role. PE in COVID-19 infection has become a mammoth challenge since the diagnosis is quite challenging due to overlapping symptoms, lack of prior-known predisposing risk factors, limited resources, and viral transmittance risk. Numerous factors arising out of the viral load or treatment lead to an increased risk for PE in COVID-19 patients, besides the fact that certain unknown risk factors may also contribute to the incidence of PE in COVID-19 patients. The management of PE in COVID-19 infection mainly comprises thromboprophylaxis and anticoagulant therapy with mechanical ventilation, depending on the risk stratification of the patient, with a post-COVID-19 management that prevents recurrent PE and complications. This review aims to discuss various aspects of COVID-19-infection-associated PE and major differential aspects from non-COVID-19 PE.

## 1. Introduction

Pulmonary embolism (PE) is the more serious clinical presentation of venous thromboembolism (VTE). It is a prevalent condition that may cause hemodynamic compromise, resulting in significant morbidity and mortality [1]. The pathophysiological events involve the obstruction of the pulmonary vasculature caused by the lodging of emboli in the pulmonary arteries. Although endogenous lysis leads to dissolution of most of the small and moderate emboli, some emboli resist lysis and persist, subsequently obturating one or more vessels. A large embolus can obstruct the pulmonary vasculature to the extent where it increases strain on the right heart, leading to hypotension and death [2]. PE is the third most common cause of cardiovascular death worldwide after stroke and heart attack [3]. PE used to be a terminal event before advances in diagnostics and treatment, and the diagnosis and management of PE is still a challenge with its multitude of symptoms and increasing number of underlying medical conditions as potential risks [4]. The treatment outcome is largely determined by the patient hemodynamic status and right ventricular overload. A clinical presentation suspected of PE requires an expeditious diagnosis and treatment to decrease the mortality and morbidity [5]. While the most common origin of thrombus in PE has been identified as emboli from proximal leg or pelvic vein thromboses, numerous conditions associated with immunocompromised patients and those with comorbidities can trigger its onset [2]. Factors that increase hypercoagulability and/or lead to immobilization are the most common established risk factors for PE. Acute infection is also an associated risk factor for PE in both hospitalized and nonhospitalized patients and has been recognized as a potent risk factor independent of immobilization [4]. Although infection is a sporadic cause of PE, the strike of the COVID-19 pandemic since December 2019 has resulted in PE being a more serious consequence as result of the severe acute respiratory syndrome coronavirus 2 (SARS-CoV-2) infection [6]. Although COVID-19 has posed a challenge in the form of the high incidence of multiorgan involvement in comparison with other viral infections, the respiratory system is one of the most commonly involved organs. [7]. The virus exaggerates the inflammatory response and can lead to severe manifestations, such as adult respiratory syndrome, sepsis, coagulopathy, and death in a proportion of patients. Although a large proportion of patients present mild symptoms, a significant proportion can progress to severe complications, such as renal and cardiac injury, disseminated intravascular coagulation, or sepsis, leading to organ failure and death [7,8]. SARS-CoV-2 infection could increase predisposition to venous and arterial thromboembolism as a result of excessive inflammation, hypoxia, immobilization, and diffuse intravascular coagulation [9]. Cui et al. (2020) reported the prevalence of VTE to be 25% [10]. Increasing data from clinical and autoptic findings the world over strongly indicate an association of COVID-19 pneumonia with massive pulmonary microembolism and pulmonary infarction as a result of a wide thrombotic response to the infection. Development of PE in COVID-19 patients is a challenging situation since the patient is in delicate equilibrium and has an already high risk of thrombosis due to other predisposing factors for thromboembolism, such as hospitalization, immobility, and corticosteroid therapy [11]. PE in COVID-19 patients seems characteristically different from PE in non-COVID-19 patients, which heightens the challenges in treatment and management. In this review, we aim to discuss the differential aspects of PE resulting from SARS-CoV-2 infection as against PE in non-COVID-19 patients and the various challenges posed by PE in the context of COVID-19.

## 2. Incidence

Prior to the strike of COVID-19, the overall annual incidence of PE was estimated to be 0.6 per 1000. It was, however, anticipated that the occurrence is higher since only 40%–50% of premortem cases were diagnosed for PE [12,13]. With the onset of COVID-19, a marked increase, about more than double, in the incidence of PE the world over has been observed [14]. COVID-19 infection has PE as the most common manifestation, with in-hospital incidence being higher in ICU patients compared with those hospitalized in general wards [9,15]. The incidence of PE has been mostly reported by sparse retrospective cohort studies that may have underestimated the incidence owing to a lack of methodical screening for this complication. Observational data suggest that 7%–39% of patients with COVID-19 infection who require mechanical ventilation have acute PE or deep vein thrombosis (DVT), and the probability of developing PE is moderate to high in those with symptoms of DVT [16]. In a Chinese study, Miesbach et al. (2020) reported that up to 40% of the patients developed PE chiefly localized in small branches of the pulmonary artery [17]. In a French study, Poissy et al. (2020) reported PE in 20.6% of the patients during their stay in the ICU, with a median time of ICU admission of 6 days [18]. In a French study, a twofold increase in the incidence of PE was observed in patients hospitalized in the ICU during the same time interval in 2019, with a similar severity score. Hauguel-Moreau et al. (2020) compared the incidence of PE during a 3-year period from January 2017 to April 2020 and observed a 97.4% increase of PE incidence as compared with that from 2017 to 2019 [19]. Another study by Bompard et al. (2020) in a French population reported a PE incidence of 50% in ICU patients, 18% in other patients, and an overall 24% cumulative incidence in patients with COVID-19 pneumonia [20]. In a Spanish study, Mestre-Gómez et al. (2020) reported the cumulative incidence of PE to be 6.4 % in COVID-19 patients [21]. Benito et al. (2020) reported a 2.6% incidence of PE in COVID-19 hospitalized patients in a Spanish study [22] (Table 1). Liao et al. (2020) reported that, overall, 15.3% of COVID-19 patients developed PE, and the incidence of PE in COVID-19 patients was higher by 3% than that in patients with seasonal and pandemic influenza [23]. Piroth et al. (2020), in their nationwide retrospective cohort study in France, found that the rate of PE was clearly higher in COVID-19 (3.4%) than in 2018–2019 seasonal influenza (0.9%) [24]. Data from numerous meta-analyses also strongly indicate a higher incidence of PE in COVID-19 patients, especially in ICU settings. Jiménez et al. (2020), in their meta-analyses that included a pooled sample of 18,093 patients, reported an overall pooled incidence of PE of 7.1% in COVID-19 patients [25]. Zhang et al. (2020), in their meta-analysis of 40 clinical studies that involved 7966 patients hospitalized with COVID-19, reported the pooled prevalence of VTE, PE, and DVT for consecutive hospitalized patients to be 13%, 8%, and 7%, respectively. ICU patients had a pooled VTE prevalence of 31%, which was significantly higher than non-ICU patients (prevalence of 7%). Compared with non-ICU patients, ICU patients also had a threefold higher pooled prevalence of PE (17% vs. 4%) and DVT (25% vs. 7%) [26]. Meta-analysis involving 27 studies with 3342 patients with COVID-19 by Suh et al. (2020) reported pooled incidence rates of PE and DVT of 16.5% and 14.8%, respectively. PE was more frequent in patients admitted to the ICU than those not admitted to the ICU (24.7% vs. 10.5%) [27,28]. Boonyawat et al. (2020), in their meta-analysis to demonstrate the pooled incidence of venous and arterial thromboembolism in COVID-19 patients in various settings, reported a pooled incidence of VTE of 28% and 10% in ICU and non-ICU settings, respectively [29]. All these studies indicate SARS-CoV-2 infection as a factor for a sudden surge in the incidence of PE the world over.

To date (January 2021), a total of over 100 million cases have been reported all over the world from the start of the outbreak, and the admission rate to the ICU varies from 3% to 100% [52]. This is highly denotive of a sharp upsurge in the incidence of PE the world over with COVID-19 infection being an imponderable factor. However, a precise rate of incidence of PE in COVID-19 still requires meticulous evaluation since interpretations may be affected by the nature, sensitivity, and specificity of the studies carried out to explore the incidence [23]. Another factor that may mask the exact incidence is the lack of reporting of PE from all the nations, especially the densely populated ones and the ones that were the epicenters. Additionally, an anticipated second wave of the pandemic, by now in a few parts of the world, will also determine the incidence of PE in COVID-19 patients. This sudden upsurge in the incidence of PE has placed a tremendous burden on the health care systems worldwide, and their unpreparedness has led to high mortality from COVID-19, despite lower case fatality rate [47].

## 3. Risk Factors

The risk factors for PE in COVID-19 patients differ from traditional thromboembolic risk factors. Numerous studies have reported them to be independent clinico-biological parameters, which are mostly driven by inflammation and coagulopathy [53,54]. The factors that have been identified as risk factors for PE in COVID-19 patients are male gender, history of stroke or atrial fibrillation, chest pain, and dyspnea. The presence of systemic inflammation, higher D-dimer level, elevated C-reactive protein level, rising D-dimer value over time, high fraction of inspired oxygen, and severe pulmonary lesions in computed tomography scan is also associated with a higher risk of PE. Another noteworthy feature is that patients with PE show extended delay from onset of symptoms to hospitalization compared with patients without PE [30,53]. The well-known conventional risk factors of thromboembolism, such as fracture of the lower limb, hospitalization for heart failure or atrial fibrillation/flutter, hip or knee replacement, major trauma, myocardial infarction, spinal cord injury, postpartum period, and obesity, did not seem to be risk factors for PE in most studies, although they may still have some relevance [30,55]. Poyiadji et al. (2020), however, reported that African Americans and obese patients are more likely to develop PE [56]. No strong association has been found between a higher risk of PE and cardiovascular comorbidities, such as diabetes, hypertension, chronic heart failure, and coronary artery disease, in various studies [53]. Certain therapeutic interventions used for the treatment of infection may also act as risk factors, if not prescribed with caution. The convalescent plasma therapy used as a treatment for COVID-19 infection in many settings itself can pose a risk since it contains procoagulant factors and can trigger PE as the patient is already having an increased prothrombotic state [57]. The necessity for mechanical ventilation in some patients can also act as a strong risk for developing PE in COVID-19 patients [32]. In most of the cases of refractory respiratory dysfunction in COVID-19, veno-venous extracorporeal membrane oxygenation (ECMO) may represent an efficient life-saving rescue therapy; however, this amplifies the risk for PE [58]. ECMO leads to systemic activation of coagulation and inflammation pathways that result from the large and continuous contact surface between the humoral and cellular components of the blood and the extracorporeal circuit, leading to increased chance of PE [59]. This can be correlated to the observational data that show that 7%–39% of patients with COVID-19 infection requiring mechanical ventilation have acute PE/DVT [16]. Since no major conventional predisposing risk factors for PE have been found to be associated with the incidence of PE in COVID-19 in most of the studies; it may indicate mild to moderate COVID-19 infections to instigate PE. This may predict of COVID-19 being an independent risk factor for PE; however, this needs validation studies [54]. The absence of conventional predicting factors for PE results in a strenuous diagnosis. This often leads to underreporting, especially with an exponentially growing number of patients and in low economic settings.

## 4. Virus-Inflected Pathophysiology

Most often, non-COVID-19-associated PE has thrombus of the lower extremities, especially the calf veins, as its origin. The thrombus sets about due to decreased blood flow and propagates because of local hypercoagulability resulting from hypoxia and hemoconcentration. A smaller percentage of emboli are developed from upper extremity veins and may have provoking risk factors as contributors. The thrombus travels from the point of origin and lodges in the pulmonary arterial system, passing through the systemic venous system and the right-sided chambers of the heart [2,60]. While an acute minor PE may not cause any serious symptoms, acute major PE reduces the cardiac output and is often fatal [57]. While PE has been reported to occur in other viral pneumonias also, the frequency is lesser compared with that in COVID-19 patients [20]. This indicates a varied pathophysiology that is modulated by the virus. It has been postulated that a unique local thrombus formation occurs primarily in the lungs because of the inflammatory process rather than emboli developing in other body parts in COVID-19-associated PE [61]. Although the exact pathophysiology of PE in COVID-19 needs validation, it is suspected to be multifactorial, being more platelet dependent, mostly associated with virus-mediated endothelial injury, and with angiotensin-converting enzyme-2 (ACE2) having a pivotal role.

The role of platelets in the pathophysiology of COVID-19-associated PE is being explored widely. This is in view of earlier sufficient studies demonstrating that viruses can directly interact with platelets and modulate their thrombotic and inflammatory functions. Accumulating data indicate an important role of platelets in COVID-19-associated thrombosis. Platelets may take up SARS-COV-2 mRNA independent of ACE2 during SARS-CoV-2 infection, while endothelial damage in COVID-19 disease may release important platelet agonists that cause overactivation of platelets [62,63]. The platelets in COVID-19 patients are reported to be hyperreactive and show increased aggregation, adhesion, and spreading on both fibrinogen and collagen. This increased platelet activation and aggregation may be partly due to increased MAPK pathway activation and thromboxane generation. Increase in the markers of platelet activation, such as P-selectin, CD63, and soluble CD40L, in COVID-19 patients is well reported. A significant increase in von Willebrand factor (VWF) in COVID-19 patients indicates a tendency towards platelet plug formation and thrombosis [64,65]. Once activated, the platelets form aggregates with neutrophils, which are frequently seen in thromboinflammation and are reported to be elevated in COVID-19 patients. Interaction between activated platelets and neutrophils also leads to the formation of neutrophil extracellular traps (NETs), which can lead to thrombus formation in lung microcirculation. Significantly increased NETs have been observed in the blood and pulmonary capillaries of COVID-19 patients, further strengthening the role of platelets in COVID-19-induced thrombosis [66,67]. In acutely ill COVID-19 patients, platelets may show a significant change in gene expression in pathways associated with protein ubiquitination, antigen presentation, and mitochondrial dysfunction. Such data indicate that platelet gene expression is altered, and functional responses are significantly increased during SARS-CoV-2 infection, which may contribute to thrombotic events in COVID-19 patients [62].

**Figure 1:**
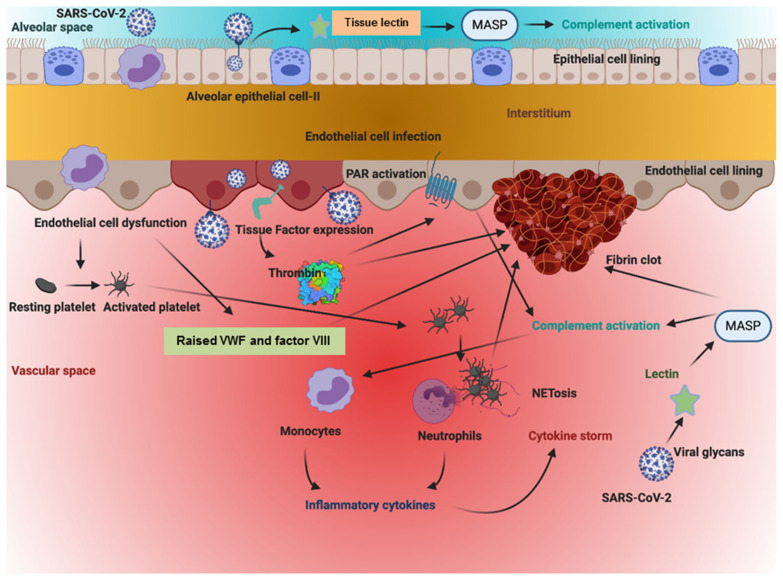
The above figure illustrates how the SARS-CoV-2 virus sets off a series of chain reactions in the intracellular and extracellular spaces of cells of the vasculature lining the alveolar space. This simultaneously sets into motion coagulation, inflammation, and complement-based pathways, resulting in a hypercoagulable state and a cytokine storm that worsens the clinical state of the individual. As is apparent from the figure, each pathway feeds into the enhancement of the other.

Endothelial injury may also be a major contributor to the pathogenesis of PE in COVID-19 patients. Currently, it is postulated that SARS-CoV-2 enters target cells through ACE2 by receptor-mediated endocytosis; however, other receptors, such as CD209L (L-SIGN), CD209 (DC-SIGN), neuropilin receptors, and CD147/basigin, may also facilitate the SARS-CoV-2 entry [68]. ARS-CoV-2 has a 10- to 20-fold increased binding affinity to ACE2 compared with SARS-CoV-1, and this high affinity may account for the increased pathogenicity of the virus [69]. ACE2 is well expressed in endothelial cells in the lungs (Figure 1), and the virus invasion may have an impact on the lung circulation through the renin–angiotensin system. Once ACE2 binds to SARS-CoV-2, it is internalized and downregulated on endothelial cells. This downregulation of ACE2 may result from ADAM-17/TACE activation by the SARS spike protein, which cleaves and releases ACE2, and/or the endocytosis of the ligand/receptor complex, followed by subsequent intracellular degradation. The degradation promotes proinflammatory and prothrombotic processes in the lung triggered by local angiotensin II (Ang II) hyperactivity [70]. Given that ACE2 is an enzyme involved in the renin–angiotensin–aldosterone system pathway, it converts Ang II into Ang (1–7), which acts on the MAS receptor. It can be perceived that a decreased ACE2 expression by endothelial cells in response to SARS-CoV-2 infection decreases the generation of Ang (1–7), which subsequently reduces the activation of MAS, consequently promoting a local prothrombotic state in endothelial cells. A downregulated ACE2 expression might also indirectly activate the kallikrein–kinin system (KKS), which leads to increases in vascular permeability [63,71]. A precise balance between the KKS and renin–angiotensin systems is necessary to regulate the thromboresistance of endothelial cells, and any disruption can have adverse outcomes [72,73].

Another major pathological mechanism that was considered crucial for PE in COVID-19 patients is “cytokine storm”; however, numerous recent studies seem to question its relevance [74,75]. The lung cells, in response to SARS-CoV-2 infection, generate proinflammatory cytokines and chemokines, attracting immune cells at the spot and further promoting inflammation and establishing a proinflammatory feedback loop. A defective immune system can lead to an overproduction of proinflammatory cytokines, causing a significant damage to the lung structure. This can cause a sustained inflammatory response, resulting in cytokine storm [76,77]. The cytokine storm involves the release of elevated plasma levels of proinflammatory cytokines, such as interleukin (IL)-2, IL-6, IL-7, IL-8, interferon gamma-induced protein 10, monocyte chemotactic protein-1, granulocyte colony-stimulating factor, macrophage inflammatory protein 1a, and tumor necrosis factor [78]. This storm leads to the development of hemophagocytic lymphohistiocytosis and activates the blood coagulation system [79]. This increases the risk of intravascular microthrombosis and secondary local consumption coagulopathy [80]. This is validated by the presence of platelet–fibrin thrombi in the small arterial vessels in lung necropsies [81]. Additionally, in an attempt to clear the viral load, white blood cell activation and clot formation increase the procoagulant state and may contribute to PE [82]. The fact that coagulation and inflammatory pathways are intricately coupled to each other has major implications for PE in COVID-19. Several hypotheses currently exist regarding the role of coagulation protein in the PE-related phenotype of COVID-19. Von Willebrand factor, an acute-phase coagulation protein, has been shown to be 5- to 10-fold elevated in symptomatic COVID-19 patients [83]. As a result, the protease ADAMTS13, which is the downregulator (through cleavage) of VWF in blood, becomes relatively deficient owing to overconsumption. This relative deficiency of ADAMTS13 can result in an altered VWF multimer pattern with larger VWF multimers which may affect the microvascular circulation in the capillaries of organs including the lung. The larger VWF multimers also can serve as an activating surface for amplification of complement pathways. VWF is also known as a risk factor for macrovascular thrombosis. VWF can have an indirect effect on thrombosis mediated by coagulation factor VIII (FVIII), since VWF is the carrier protein for FVIII and FVIII activity is dependent on VWF levels. FVIII is a strong acute-phase protein. FVIII activities are increased four to six times in COVID-19 patients [83]. Raised FVIII/VWF levels of >150% are known to be significant risk factors for thrombosis [84]. Hyperactivation of FVIII can result in a thrombin burst, creating a prothrombotic scenario. FVIII is downregulated by the activated protein C (APC) system. Excessive FVIII activity may exhaust the APC system and result in a very significant risk for thrombosis in macrovascular circulation and thus contribute to the high incidence of primary lung embolism in patients with COVID-19 disease. Another critical point to consider when talking about the links between COVID-19, coagulation, and PE is the ABO blood group [85]. The ABO blood group has a significant influence on VWF and FVIII activity. The blood groups O and A2 have been shown to have 50% lower VWF and FVIII activity than blood groups A1, B, A1B, and A2B [86]. Therefore, they might have a secondary but indirect effect on COVID-19 pathophysiology mediated by VWF and FVIII. The heterogeneity and overlapping in the mechanisms of PE in COVID-19 may pose a challenge in defining a line of treatment and further management to reduce complications especially when choosing the nature of complication. This is of significant concern since PE survivors have a higher risk of developing post-PE syndrome and recurrent PE and other functional impairments. However, more detailed investigations are needed to define the multiple ways in which COVID-19 manifests into a hypercoagulant pathophysiology in PE.

## 5. Diagnosis

Establishing a precise diagnosis of PE has often been challenging and quite difficult. This is because of the nonspecific symptoms of PE and/or the absence of any symptoms at all [87]. Pre-existing cardiac and pulmonary conditions further make the diagnosis difficult [60]. However, an early diagnosis is highly recommended to improve prognosis and requires an interdisciplinary approach to avoid misinterpretations. Although diagnostic strategies differ in various clinical settings, the diagnostic workup includes clinical probability assessment, followed by biomarker assessment and imaging. The Wells Clinical Prediction Rule is most commonly used to predict possibility and validates the clinical probability of low or intermediate risk and high risk [88]. A low or intermediate probability of PE needs D-dimer measurements, followed by an imaging test, while for a high probability of PE, imaging tests are mandatory [89]. Imaging tests predominantly have computed tomography pulmonary angiography (CTPA) as gold standard [90]. The diagnosis of COVID-19-infection-associated PE is further complicated, and an altered approach is rendered. Primarily, in COVID-19 patients, the signs and symptoms of acute respiratory distress syndrome and PE may overlap, escalating the diagnostic challenge. Secondly, other underlying conditions, such as pregnancy and cardiopulmonary comorbidities, further amplify the diagnostic difficulties. Another major hurdle in opportune diagnosis is strict isolation precautions that require minimum contact of health-care workers with the patient [90]. This has led to a change in the diagnostic approach to attain the best possible diagnosis with the least risk to the health-care staff and patient. A multidisciplinary approach is still indispensable but is more customized for patients. The physician evaluating COVID-19 patients should keenly estimate the progress of COVID-19 symptoms along with the risk factors for PE. Testing for PE should be prioritized if the patient exhibits hemodynamic instability or an unexplained poor gas exchange for the stage or presents with minimal pulmonary infiltrates or signs of acute right ventricular (RV) overload [91,92]. Studies from France suggest that PE occurred in COVID-19 patients at a median of 6 days [18]. As a marker for hemostasis, D-dimer testing has been followed almost the world over; however, necessitating D-dimer in diagnostic decision-making for PE has an overall varied response [93,94]. Normal levels of D-dimer have the ability to rule out PE, especially in the context of low pretest probability, and can annul radiological imaging. However, a false negative rate of D-dimer testing, although very rare, cannot be ruled out [16]. Elevated D-dimer (>2.0 µg/mL) levels have been associated with worst outcomes and increased mortality in COVID-19 patients all over the world [20]. Vidali et al. (2020), in their study to estimate D-dimer as an indicator of prognosis in SARS-CoV-2 infection, evaluated 16 studies and found 13 studies to have significantly increased D-dimer in COVID-19 patients compared with healthy controls. The dimer was also increased in COVID-19 patients with severe disease compared with those with nonsevere disease [95]. D-dimer level excludes very few patients from the need for a confirmatory imaging test. If the D-dimer levels change from normal to high or are rapidly increasing, then imaging is required. However, other factors that interfere with D-dimer levels, such as secondary infection, myocardial infarction, renal failure, and coagulopathy, also need to be screened [16]. The necessity of imaging, especially CTPA, in the detection of PE in COVID-19 patients presents one of the major challenges. The transfer of a patient to the CTPA suite increases the chances of disease transmission and calls for disinfection of the CT suite. The use of contrast media might be contraindicated in patients with severe COVID-19 with associated renal dysfunction or patients with contrast allergy [96]. Strict isolation measures because of the chances of virus aerosolization and the possibility of unavailability of personal protective equipment further limit CTPA in a pandemic setting. To overcome this limitation, it is highly recommended that bedside echocardiogram and lower-extremity ultrasonography be the preferred modalities [92]. Echocardiography remains the first-line imaging modality for early detection of PE in the assessment of the critically ill. Echocardiography provides some valuable diagnostic information, such as noninvasive estimation of cardiac output, diastolic function parameters, and evaluation of right ventricular function and pulmonary circulation, which is highly valuable in patients that have severe hypoxemia and require mechanical ventilation. All these parameters are helpful in treating critically ill COVID-19 patients, especially as regards optimizing positive end-expiratory pressure and the possibility of successful weaning from mechanical ventilation [97]. When carried out, balancing the benefits and risks of disease transmission, echocardiography findings can lead to a treatment change in up to 33% of such patients [98]. Echocardiography can be particularly helpful in the management of selected COVID-19 patients, especially those with elevated D-dimer and troponin levels, and can be helpful in changing the treatment regime to improve the outcomes. Scudiero et al. (2020) reported an echocardiography-induced change in the treatment of 16% of the COVID-19 patients in their study. A higher systolic pulmonary arterial pressure and documented cardiac thrombi on echocardiography were associated with treatment changes in these patients. They observed significantly (*p* = 0.010) higher rates of mortality and cardiogenic shock in patients with PE as compared with non-PE patients. In their study, tricuspid annular plane systolic excursion and systolic pulmonary arterial pressure were the only parameters that were independently associated with the occurrence of PE in COVID-19 patients [99].

## 6. Prophylactic and Therapeutic Interventions

The fundamentals of therapy for PE in non-COVID-19 settings are the prevention of new embolic episodes by employing anticoagulant treatment or a filter in the inferior vena cava [100]. The treatment of PE in COVID-19 patients, however, deems both prophylactic and curative therapies to be vital, depending on the level of risk and the condition of the patient [92] (Table 2). While numerous guidelines have been issued by various bodies, the responsibility of health professionals to make appropriate decisions in the circumstances of individual patients remains prime. The International Society on Thrombosis and Haemostasis (ISTH) and the American Society of Hematology (ASH) recommend a prophylactic dose of LMWH (40 mg qd) or subcutaneous unfractionated heparin (5000 IU tid) for COVID-19 patients; however, the precise effective dose still needs to be determined with considerations for bleeding disorders [17,101,102].

In high-risk patients with no initial diagnosis of PE, anticoagulant therapy may be empirically considered if they rapidly develop physico-clinical symptoms in accord with PE [102]. For confirmed PE in COVID-19 patients, administration of systemic anticoagulant therapy is highly recommended by the European Society of Cardiology [103]. However, this requires a thorough evaluation of contraindications, primarily those concerning bleeding. The precise dose of administration is evaluated as per the treatment regime of the institute and the treatment team. Parenteral therapy with LMWH and unfractionated heparin are the most commonly used anticoagulants in PE for COVID-19 patients [92]. LMWH is preferred in the current demanding situation owing to its cost-effectiveness and ease of availability [104]. The use of unfractionated heparin as an experimental therapy is strongly associated with improved survival in patients with markedly elevated D-dimer levels [105]. The therapy has the twofold advantage of having cytoprotective and antiviral effects besides having anticoagulant property [106]. Unfractionated heparin limits the fibrin deposition and microthrombus formation and is employed to treat systemic prothrombotic complications; however, clearance of fibrin clusters in the alveolar space cannot be achieved [107]. Perceptive monitoring of the patient is required to derive a therapeutically functional dose for unfractionated heparin therapy, while LMWH is used widely at a dose of 40 mg or higher and requires no cautious monitoring [108]. Another beneficial aspect of LMWH therapy is a low tendency to induce bleeding and thrombocytopenia compared with UFH [106]. A delay or failure in the detection of heparin-induced thrombocytopenia is associated with poor outcomes of the treatment [109]. In patients with known heparin-induced thrombocytopenia, fondaparinux is the therapeutic agent of choice, besides argatroban being also administered widely [109,110]. It is being targeted by various expert groups to reach a dose beyond the standard dose to attain maximum benefit while overcoming the limitations of the standard dose. Even though standard pharmacological thromboprophylaxis is recommended in hospitalized patients, various expert groups have proposed to increase the dose of anticoagulants in critically ill patients with COVID-19. Tacquard et al. (2021) in their trial reported a higher prophylactic anticoagulation dose to be significantly associated with a reduced risk of thrombotic complications. Cumulative exposure to a higher prophylactic anticoagulant dose, however, was not associated with reduced mortality on day 14 and not associated with increased bleeding risk compared with standard dosing [111].

In a randomized controlled trial involving seriously ill COVID-19 patients, Bikdeli et al. (2020) aimed to infer whether an intermediate dose of anticoagulants apart from the standard dose could impart a benefit by decreasing the thrombotic event rates. An intermediate dose was thought to have the potential to confer a benefit while mitigating the high risk of bleeding associated with higher doses of therapeutic anticoagulation. The results from such trials can be of considerable importance in determining the efficacy of intermediate doses of anticoagulants [112].

Patients eligible for oral anticoagulants are preferred direct oral anticoagulant (DOAC) over a vitamin K antagonist, although warfarin or acenocoumarol are still used in numerous settings [113]. Recommendation of DOACs is possible only in patients with stable hemodynamics after an acute phase. High caution is recommended for a shift from vitamin K antagonists to DOACs in a presently pressing situation to avoid complications arising from erroneous change, leading to thromboembolism or bleeding [114]. A serious evaluation of drug–drug interactions of DOACs with antiviral therapies and the estimation of the risk of hepatic and renal toxicity are highly imperative in the prescription of DOACs. DOACs, when started after eliminating contraindications, bear a low-impact viral transmission for health-care personnel as regular and frequent blood tests are not required and abate heparin pseudo-resistance [113,115]. DOACs highly favor a timely discharge from the hospital but concurrently bear a risk of organ dysfunction, which may not be reversed in certain setups [116]. Although there are already existing practice guidelines for the recommended use of DOACs for patients with acute symptomatic VTE, different suggestions for the preferred anticoagulant in patients with COVID-19, particularly for the critically ill, have been made. The various recommendations made by the American College of Chest Physicians (CHEST) panel and other organizations after achieving a consensus on the use of DOACs in acutely and critically ill COVID-19 patients are as follows: (i) in acutely ill hospitalized patients with COVID-19, anticoagulant thromboprophylaxis with LMWH, fondaparinux, or UFH is recommended over anticoagulant thromboprophylaxis with a DOAC. The panel warns against the use of DOACs in these patients because of the high risk of rapid clinical deterioration in these patients. In addition, it is possible that most of these patients may be receiving antiviral agents or other investigational treatments that may significantly affect the pharmacodynamics of DOACs and hence increase the bleeding risk associated with the DOACs. (ii) In critically ill patients with COVID-19, the panel recommends anticoagulant thromboprophylaxis with LMWH or UFH over anticoagulant thromboprophylaxis with fondaparinux or a DOAC. The panel strongly warns against the use of DOACs in critically ill patients secondary to the high likelihood of drug–drug interactions, high possibility of acute kidney injury in these patients, and their hemodynamic instability. (iii) COVID-19 outpatients receiving warfarin who are unable to get international normalized ratio monitoring because of isolation may have DOACs initiated. The American College of Cardiology considers it reasonable to extend prophylaxis with LMWH or DOACs for up to 45 days in patients at a high risk of VTE and low risk of bleeding [92,115].

Thrombolytic therapy is conventionally reserved for COVID-19 patients with confirmed PE who are experiencing hemodynamic instability or hemodynamic collapse, with limited recommendation for hemodynamically stable patients [92,117]. Thrombolytic therapy has an overall limited efficacy in hemodynamically stable patients, and evidence does not strongly validate the treatment of COVID-19 pulmonary microthrombi in such patients. The therapy highly predisposes one to adverse events, while indications and contraindications for thrombolysis remain unchanged [117]. Systemic thrombolysis may be a preferred option for patients who are appropriate candidates for advanced therapy but unattainable for an invasive approach due to limited resources or viral transmission risks. Philippe et al. (2021) reported that systemic thrombolysis is highly effective in PE with right ventricular (RV) failure in patients with COVID-19 suffering from acute respiratory distress syndrome, with no cardiogenic shock. They reported seven patients; three had high-risk PE, and four had intermediate high-risk PE as per the European Society of Cardiology (ESC) severity scale. The systemic thrombolysis (recombinant tissue plasminogen activator) in the patients according to the standard protocol of 10 mg for over 15 min and then 90 mg for over 120 min was found to be associated with improved treatment outcomes [118]. Five patients had a reduction of the Brescia-COVID Respiratory Severity Scale besides a decrease of the RV dysfunction. They reported no major bleeding events after the thrombolysis; however, the mortality after systemic thrombolysis was up to three of seven patients. In a COVID-19 hospitalized patient presenting high-risk PE related to a right heart thrombus, Scudiero et al. (2020) found systemic thrombolysis to be highly effective and recommend the therapy for life-threatening PE in COVID-19 patients [119]. It is highly preferred to limit the use of catheter-directed therapies during the current outbreak unless warranted by critical situations to limit viral transmittance since inconsiderable data support lower mortality from routine use of advanced therapies [116,120]. Mechanical thromboprophylaxis becomes indispensable when pharmacological thromboprophylaxis is contraindicated especially in immobilized patients [116,121]. Pneumatic compression devices are most widely recommended as per the major societal recommendations and guidelines addressing the management of coagulopathy in COVID-19 patients and WHO [121,122]. A combined pharmacological and mechanical prophylaxis is not recommended unless the patient has a worsening trajectory and there is no contraindication for each modality [123]. Extracorporeal membrane oxygenation (ECMO) with catheter-directed treatment or surgical embolectomy is preferred in case of cardiac arrest or refractory shock to prevent worse outcomes. ECMO therapy needs to be given strictly following the existing anticoagulation guidelines to prevent thrombotic complications arising from the activation of the coagulation pathways due to the therapy itself, considering the hypercoagulable status of COVID-19 patients.

## 7. Post-COVID-19 Thromboprophylaxis

After discharge, VTE prophylaxis is not recommended for COVID-19 patients [124]. However, in patients treated for COVID-19 and predisposed moderately or severely to PE, a computed tomography pulmonary angiogram (CT-PE) within 1 month of being negative for COVID-19 may be recommended, if they had not undergone any diagnostic imaging due to the COVID-19 surge. In patients with a high risk of PE, a follow-up and 3 months of thromboprophylaxis may be recommended [110]. The Food and Drug Administration has approved rivaroxaban at 10 mg daily for 31 to 39 days in such patients [125]. Telemedicine is an expedient method that can provide follow-up care to such vulnerable patients with minimal risk of viral transmission [126]. Hospitalized patients, when getting discharged and apparently seeming stable and asymptomatic, may still have the procoagulant effect of COVID-19 extended by a few weeks. This, combined with less mobility during hospitalization and other complications, puts the patient at a high suspicion of recurrent PE after discharge [127,128]. It is evident from retrospective observational cohort studies that COVID-19 patients discharged from the hospital without anticoagulation had a cumulative incidence of thrombosis (2.5%), VTE (0.6%), and major hemorrhage (0.7%) with clinically relevant non-major bleeds (2.9%) on day 30 following discharge [129]. The prevention of a repeated episode requires extending an outpatient VTE prophylaxis; however, the decision needs to balance the reduced risk of VTE with the risk of increased bleeding events. LMWH is preferred to DOAC due to potential drug–drug interactions and/or frequent comorbidities [102,103,104,105,106,107,108,109,110,111,112,113,114,115,116,117,118,119,120,121].

## 8. Mortality

Prior to the strike of the COVID-19 pandemic, time trend analyses suggested that case fatality rates of PE were decreasing in European, Asian, and North American populations over the last 15 years [98,99,100,101,102,103,104,105,106,107,108,109,110,111,112,113,114,115,116,117]. However, PE in COVID-19 patients is associated with clinical worsening of the disease outcome and increased mortality rate. Numerous studies strongly suggest PE in COVID-19 patients as an increased risk factor for mortality. Liao et al. (2020) reported a substantially high mortality rate (45%) for COVID-19 patients with PE when compared with COVID-19 patients with no PE [23]. Bompard et al. (2020) also reported a higher mortality rate in a PE-positive group (13%) than in a PE-negative group (12%) [20]. With the cumulative incidence of death due to COVID-19 being about 1.8 million, the contribution of PE to mortality necessitates that first-line healthcare providers be cautious about the incidence of PE and its associated complications in COVID-19 patients [130] (Table 1).

## 9. Conclusions

COVID-19 has led to enormous human casualties, with PE being a major manifestation of the infection that increases the chance of worst outcomes of the disease and increases mortality. COVID-19-associated PE patients have increased the challenge to the health-care system and care providers. Overlapping symptoms with no well-known prior risk factors lead to undiagnosed PE in COVID-19 patients, especially in settings with limited resources. The differential characteristics of COVID-19-related PE and classical PE pose a challenge to treatment since patients have varied clinico-biological presentations. It requires a thorough evaluation of the risk stratification and course of disease to design a treatment regime, with careful assessment of contraindications for every patient. This calls for a multidisciplinary approach, which is constrained due to the overburden of patients and a high risk of viral transmittance. Although numerous guidelines have been issued to manage the condition, success has not been achieved to overcome its detrimental effects. This calls for more dedication in taking preventive measures to avoid viral exposure by the public in general and high-risk groups in particular.

## Figures and Tables

**Table 1:** Incidence and mortality of PE in COVID-19 patients.

	Author	Study Design	Mean Age (Years)	Number of Patients	PE Incidence (%)	ICU (%)	Mortality (%)	Thromboprophylaxis	PE Imaging Test
1	Artifoni et al. [30]	Retrospective cohort double-center study	64	133	10	12.78	14.28	Heparin (unspecified)	Low limb venous duplex ultrasonography/CTPA *
2	Lodigiani et al. [31]	Retrospective single-center	66	388	2.8	16	26	Low-molecular-weight heparin	Two-point compression ultrasonography
3	Poissy et al. [18]	Retrospective single-center	57	107	20.6	20.6	14	Unfractionated heparin or low-molecular-weight heparin	CTPA
4	Grillet et al. [32]	Retrospective single-center	66	100	23	39	NR	NR	CTPA
5	Leonard-Lorant et al. [33]	Retrospective double-center	63.5	106	30.2	75	NR	Low-molecular-weight heparin	CTPA
6	Llitjos et al. [34]	Retrospective double-center	68	26	23.1	An ICU study	12	Low-molecular-weight heparin or unfractionated heparin	Complete duplex ultrasound
7	Klok et al. [9]	Retrospective multicenter	64	184	35.5	An ICU study	22	Low-molecular-weight heparins	CTPA and/or ultrasonography
8	Thomas et al. [35]	Retrospective single-center	59	63	7.9	An ICUstudy	16	Unfractionated heparin/low-molecular-weight heparin	Lower limb ultrasound dopplers/CTPA
9	Middeldorp et al. [36]	Retrospective single-center	61	198	6.6	38	19	Low-molecular-weight heparin	CTPA
10	Helms et al. [37]	Prospective multicenter	63	150	16.7	An ICU study	9	Unfractionated heparin/low-molecular-weight heparin	CTPA/abdomen and pelvis CT
11	Hékimian et al. [38]	Retrospective single-center	NA	51	16	An ICU study	NR	NR	CTPA
12	Galeano-Valle et al. [39]	Prospective observational single-center	64.3	24	45.8	3	NR	NR	CTPA
13	Bompard et al. [20]	Retrospective double-center	64	135	23.7	17	12	Heparin (unspecified)	CTPA
14	Soumagne et al. [40]	Retrospective multicenter	63.5	375	15	An ICU study	NR	Anticoagulant not specified	NR
15	Fraissé et al. [41]	Retrospective single-center	61	93	49	An ICU study	41	Anticoagulant not specified	NR
16	Freund et al. [42]	Retrospective multicenter	61.	3253	15	0	NR	NR	CTPA
17	Chen et al. [43]	Retrospective single-center	65	25	40	NR	24	NR	CTPA
18	Longchamp et al. [44]	Retrospective single-center	68	25	20.0	An ICU study	20	Heparin (unspecified)	CTPA/CUS **
19	Whyte et al. [45]	Retrospective single-center	61.5	1477	37	15	16	Unfractionated heparin/low-molecular-weight heparin	CTPA
20	Marone et al. [46]	Retrospective single-center	NA	101	23.7	NR	NR	Low-molecular-weight heparin	Duplex ultrasound/CTPA
21	Fauvel et al. [47]	Retrospective multicenter	64	1240	8.3	14.9	12.2	Unfractionated heparin/low-molecular-weight heparin	CTPA
22	Van den Heuvel et al. [48]	Retrospective single-center	63	51	18	37	1	NR	CTPA
23	Mestre-Gómez et al. [21]	Retrospective single-center	65	29	6.4	6.9	3.4	Low-molecular-weight heparin	CTPA
24	Faggiano et al. [49]	Retrospective single-center	70.3	25	7	NR	14	Unfractionated or low-molecular-weight heparin	CTPA
25	Gervaise et al. [50]	Retrospective single-center	62.3	72	18	57	15	NR	CTPA
26	Trimaille et al. [51]	Retrospective single-center	62.2	289	14.5	72	47	Unfractionated heparin/low-molecular-weight heparin	CTPA

* CTPA—Computed tomography pulmonary angiogram; ** CUS—Compression ultrasound.

**Table 2:** Recommendations for treatment and follow-up for patients suspected of/confirmed for PE. [90,92].

Condition of the Patient	Level of Risk for PE	Treatment
Stable	Mild and moderate risk	Pharmacological prevention is prescribed. LMWH is recommended as first-line treatment, in the absence of contraindication.
Traumatic	Mild and moderate risk	Pharmacological prevention is prescribed. LMWH is recommended as first-line treatment, in the absence of contraindication. In case of contraindication for pharmacological thromboprophylaxis, mechanical thromboprophylaxis is preferred.
Stable (discharged)	Persistent mild and moderate risk	Prolonged outpatient VTE * prophylaxis care is considered with LMWH ** over DOAC *** use to prevent drug–drug interactions, and/or frequent comorbidities.
Acute	Confirmed	Initial parenteral anticoagulation with LMWH or UFH, overlapped with latter vitamin K antagonist therapy.
Shock/hypotension	Confirmed	Systemically administered thrombolytics if there is no high risk of bleeding.
Acute/cardiopulmonary deterioration	Confirmed	Systemic thrombolytic therapy after an initiation of anticoagulant therapy in the absence of hypotension and a low risk of bleeding.
Acute	Confirmed	Systemic thrombolysis for patients eligible for advanced therapy but lack an invasive approach due to limited resources or high risk of viral transmission.
Critical (refractory circulatory collapse or cardiac arrest)	Confirmed	ECMO **** in combination with surgical embolectomy or catheter-directed treatment.
Stable (outpatient)	Confirmed	Parenteral anticoagulants overlapped with vitamin K antagonists.

* VTE—Venous thromboembolism; ** LMWH—Low-molecular-weight heparins; *** DOAC—Direct Oral Anticoagulant; **** ECMO—Extracorporeal membrane oxygenation.
